# Bisphenol A attenuates testosterone synthesis via increasing apolipoprotein A1-mediated reverse cholesterol transport in mice

**DOI:** 10.3389/fendo.2025.1514105

**Published:** 2025-01-28

**Authors:** Tong Zhao, Wenzhe Yang, Feilong Pan, Jinhao Wang, Wenqi Shao, Fangfang Chen, Kexiang Liu, Shuchen Zhao, Lijia Zhao

**Affiliations:** ^1^ College of Veterinary Medicine, Northeast Agricultural University, Harbin, Heilongjiang, China; ^2^ Key Laboratory of the Provincial Education Department of Heilongjiang for Common Animal Disease Prevention and Treatment, Harbin, Heilongjiang, China

**Keywords:** bisphenol A, apolipoprotein A1, cholesterol, reverse cholesterol transport, testosterone, lipid droplet

## Abstract

Bisphenol A (BPA), a widely used chemical compound in plastic manufacturing, has become ubiquitous in the environment. Previous studies have highlighted its adverse effects on reproductive function, as BPA exposure reduces testosterone levels. Cholesterol is involved in testosterone synthesis in Leydig cells. However, research on the mechanisms by which BPA affects testosterone synthesis from the perspective of reverse cholesterol transport (RCT) remains limited. This study aimed to investigate the effects of BPA on cholesterol levels, lipid droplet accumulation, and testosterone synthesis in TM3 cells and mice via Apolipoprotein A1 (APOA1)-mediated RCT. Adult male mice were treated by intraperitoneal injection of corn oil containing BPA (20 mg/kg) for 7 days. Testes were collected for protein extraction, RNA extraction, Oil red O staining or for Biochemical analysis. Serums were collected for detection of testosterone levels. flow cytometry, CCK8 assay, immunofluorescence or Filipin III staining was used to detect the effect of BPA on the TM3 cells. It was observed that serum and testicular testosterone levels were drastically reduced in BPA-treated mice. Moreover, lipid droplets accumulation and testicular total (TC) and free cholesterol (FC) levels were reduced in the mouse testes. Conversely, testicular high-density lipoprotein (HDL) content was partially elevated. Furthermore, BPA markedly enhanced *Apoa1* mRNA and protein expression in the mouse model. Notably, BPA significantly upregulated *Apoa1* mRNA and protein level, reduced cholesterol levels and lipid droplets accumulation, and attenuated testosterone synthesis in TM3 cells. In addition, exogenous supplement with 22-hydoxycholesterol promoted testosterone synthesis and alleviated the inhibitory effect of BPA on testosterone synthesis. Taken together, these results suggest that BPA upregulates APOA1 expression, enhances RCT, and ultimately reduces TC and FC levels in the testis. This cholesterol reduction likely led to testosterone synthesis disorders in the model, indicating that BPA inhibits testosterone synthesis in mice by disrupting cholesterol transport.

## 1 Introduction

Bisphenol A (BPA), one of the most widely used synthetic chemicals in the plastic manufacturing industry, is used in a variety of applications, including household products, medical devices, architectural glass, electronics, and industrial equipment (1, 2). More than 8 billion pounds of BPA are produced globally each year, with an annual growth rate ranging between 6–10%. Approximately 100 tons of BPA are released into the atmosphere each year, with an increasing trend from year to year (3). Moreover, BPA is the predominant and most frequently detected bisphenol (BP) in river, lake and sea water, with concentrations reaching up to 85.5 μg/L (4, 5). Due to its widespread distribution, BPA is found in the environment concentrations that can pose risks to many plant, invertebrate, and vertebrate species. BPA can be absorbed through various routes, including the human digestive tract, respiratory tract, and skin (6, 7). It accumulates in several tissues and organs and is potentially harmful to human health via different molecular mechanisms (8). Studies have also reported the presence of BPA in placental tissue, amniotic fluid, cord blood, breast milk, and follicular fluid (9, 10). Due to its biomagnification effect, BPA in the environment is ingested by organisms, passes down the food chain, and gradually accumulates in organisms, further exacerbating the safety risks faced by biological communities. A growing number of studies have shown that BPA exposure can cause disorders of the endocrine system, reproductive system, cardiovascular system, metabolism, and neurobehavior (11–14).

BPA is an endocrine disruptor that can negatively affect the male reproductive system by altering endocrine functions through various pathways (15, 16). Studies have shown that exposure of female rats to BPA leads to reproductive dysfunction in male offspring, including impaired spermatogenesis, reduced epididymal sperm counts, and decreased serum testosterone levels (17, 18). Additionally, BPA has been suggested to reduce sperm quality in goldfish by downregulating levels of testosterone and 11-ketotestosterone (19). Moreover, evidence has also shown that BPA can cause reproductive abnormalities in relation to lipid metabolism disorders in men (20). Previous studies have indicated that BPA induces an increase in the transport of fatty acids from adipose tissue to the liver, stimulating the hepatic synthesis of endogenous fatty acids, which ultimately leads to the excessive accumulation of triglycerides in the liver and, thus, a disorder of lipid metabolism in male mice (21). A previous study demonstrated that BPA exposure leads to lower cholesterol levels in zebrafish testes (22). However, few studies have investigated whether BPA regulates testosterone synthesis in mouse testes by enhancing reverse cholesterol transport (RCT).

Apolipoproteins play a key role in the transport of lipids in the body and are involved in the reverse cholesterol transport process by assisting in the transport of cholesterol, triglycerides and other lipids from one tissue or cell to another. Moreover, they regulate triglyceride levels to meet the body’s energy requirements, lipid storage, and lipid homeostasis (23). The apolipoprotein family includes Apolipoprotein A1 (APOA1), Apolipoprotein A2 (APOA2), Apolipoprotein A5 (APOA5), Apolipoprotein E (APOE), Apolipoprotein C3 (APOC3), and other major proteins. APOA1, a key protein of high-density lipoprotein (HDL), plays a crucial role in RCT and has been confirmed to have anti-atherogenic effects in the past several decades. Studies have shown that mice with APOA1-deficiency gained more body weight and fat mass than wild-type WT mice (24). Conversely, mice overexpressing *Apoa1* exhibit increased levels of lipolytic enzymes, reflecting enhanced triglyceride hydrolysis and free fatty acid oxidation within the adipose tissue (25). APOA2 is the second most abundant apolipoprotein in HDL particles and exists as a homodimer in human plasma. APOA2 concentration plays a key role in the modulation of HDL size and morphology (26). APOC3 inhibits the catabolism of triacylglycerols (TAG) from chylomicrons and very low-density lipoprotein by lipoprotein lipase and inhibits the hepatic uptake of residual lipoproteins. All the aforementioned apolipoproteins have been verified to exert vital effects on modulating lipid metabolism and homeostasis in both plasma and cells.

Cholesterol is an important member of the lipid family (27). Disruption of cellular cholesterol homeostasis can lead to a variety of pathological conditions, including atherosclerosis and Tangier’s disease (28). Cholesterol balance disorders are also associated with reproductive abnormalities (29, 30). Testosterone is a steroid hormone that plays an important role in physiological processes such as male growth and development, libido maintenance, and reproductive function (31). Cholesterol, a precursor of testosterone, is involved in testosterone synthesis in Leydig cells (32). It has been shown that cholesterol levels are positively correlated with testosterone levels (33). Thus, the availability of sufficient cholesterol to Leydig cells is a prerequisite for the production and maintenance of testosterone. Cholesterol in the testes is largely derived from endogenous synthesis and uptake of blood lipoproteins, with excess cholesterol being transported back to hepatic tissues via an RCT mechanism for eventual excretion into the bile (34). The RCT has long been considered the only pathway that transports excess cholesterol from cells to the liver for excretion (35). The critical role of APOA1 in HDL biosynthesis and RCT has been previously demonstrated (19, 36), as APOA1 binds to cholesterol via ATP-binding cassette transporter protein A1 (ABCA1) to generate discoidal (d) HDL, conversion of cholesterol carried in dHDL to cholesteryl esters (CEs) by lecithi-cholesterol acyltransferase (LCAT), matures it into its spherical form. This spherical form is then acted upon by several proteins in plasma that alter its composition and size. Then CE carried within HDL are delivered to the liver through the scavenger receptor, class B type 1 (SR-B1), where they are either utilized or excreted in the bile with the help of heteromeric dimer composed of ATB-binding cassette transporter G5 and G8 (ABCG5/ABCG8) (35, 37). Therefore, investigating the decrease in testosterone synthesis in Leydig cells from the perspective of cholesterol is advantageous. Based on the aforementioned evidence, it is postulated that BPA might inhibit testosterone synthesis by perturbing RCT in mice.

The present study aims to investigate the effects of BPA exposure on the expression of genes and proteins related to cholesterol transport using *in vivo* and *in vitro* experiments. In addition, lipid droplet accumulation and free cholesterol (FC) levels in TM3 cells and testes, as well as changes in testosterone synthesis will be assessed. Moreover, exogenous supplement of 22-hydoxycholesterol (22-OH-Chol) were used to uncover the mechanisms by which BPA increases APOA1-mediated RCT led to inhibition of testosterone synthesis in TM3 cells in the presence of BPA. This study will provide a new perspective on the mechanism through which BPA inhibits testosterone synthesis in mice by focusing on lipid metabolism.

## 2 Materials and methods

### 2.1 Chemicals and reagents

BPA (chemical purity 99%) was obtained from Sigma-Aldrich (St. Louis, MO, USA). DMEM/HIGH GLUCOSE medium, penicillin, and streptomycin were purchased from HyClone (Logan, Utah, USA). Fetal bovine serum (FBS) was purchased from ZETA™ (Shanghai, China). RNAiso Plus and PrimeScript™RT Reagent Kit with gDNA Eraser were obtained from TaKaRa (Osaka, Japan). SYBR^®^ Green Realtime PCR Master Mix were obtained from TOYOBO (Osaka, Japan). Dimethyl sulfoxide (DMSO; cell culture grade), polyvinylidene difluoride (PVDF), Oil Red O Saturated Solution 0.5%, Mayer’s Hematoxylin stain, Annexin V-FITC Apoptosis Detection Kit, Free Cholestenone Content Assay Kit and Horseradish peroxidase-linked secondary antibodies were purchased from Solarbio (Beijing, China). Enhanced Cell Counting Kit-8 (CCK-8), BeyoECL Plus, Enhanced BCA Protein Assay Kit, and sodium dodecyl-sulfate polyacrylamide gel electrophoresis (SDS-PAGE) sample loading buffer were obtained from Beyotime (Shanghai, China). APOA1 antibody was obtained from Proteintech (Chicago, Kentucky, USA). Anti-antibody coated bacteria test (ACTB) mouse monoclonal antibody was purchased from Sangon Biotech (Shanghai, China). Goat Anti-Rabbit IgG H&L/AF488 antibody was obtained from Bioss (Beijing, China). The immunostaining permeabilization buffer containing Triton X-100, bovine albumin, DAPI staining solution, and antifade mounting medium for fluorescence (with DAPI) were obtained from Biosharp (Anhui, China). Testosterone, HDL cholesterol, and total cholesterol (TC) assay kits were obtained from Jiancheng (Nanjing, China). 22-hydroxycholesterol and filipin III were obtained from Cayman Chemical (Ann Arbor, MI, USA).

### 2.2 Animals and treatments

Twelve 8-week-old male Kunming mice were obtained from The 2^nd^ Affiliated Hospital of Harbin Medical University (Harbin, China). Before the formal experiment, the mice were acclimatized for one week in a standard environment (25°C, 50% humidity, 12 h/12 h light-dark cycle) with food and water *ad libitum*. All procedures for the care and use of animals followed the guidelines of Northeast Agricultural University (NEAUEC20).

After adaptive feeding, the mice were randomly classified into two groups including control (CON) and BPA-treated group, with 6 mice in each group. The mice in the treatment group were injected intraperitoneally with BPA at a dose of 20 mg/kg of body weight daily for seven consecutive days (BPA group). The mice in the CON group were intraperitoneally injected with the same volume of corn oil. BPA was dissolved in corn oil by intraperitoneal injection. The chosen dose of 20 mg/kg BPA and 7 days treatment duration were based on previous studies (38, 39), which reported no significant signs of toxicity at this concentration, although adverse effects on the reproductive system were observed (39). Finally, the mice were euthanized by cervical dislocation, and the bilateral testes of each animal were collected. The left testis tissues were fixed with 4% paraformaldehyde overnight, the right testis tissues were stored at –80°C immediately after collection. Before euthanasia, blood samples were drawn by extirpating the eyeballs and centrifuged for serum preparation at 2000 xg for 15 min to assess serum testosterone levels.

### 2.3 Cell culture

TM3 cell, a mouse Leydig cell line, was purchased from the Cell Bank of the Chinese Academy of Sciences (Shanghai, China), which were seeded in 35-mm collagen-coated dishes and cultured in high-glucose DMEM supplemented with 10% FBS and 1% penicillin/streptomycin and maintained at 37°C in an incubator humidified with 5% CO_2_ for 24 h before treatment.

### 2.4 Cells viability assay

TM3 cells were seeded at a density of 5×10^3^ per well in 96-well plates and cultured for 24 h. BPA was solubilized by DMSO and then diluted to the appropriate concentration with cell culture solution according to the BPA concentration range reported by Li and colleagues (39), cells were treated with 0, 5, 10, 20, 40, 60, 80 and 100 μmol/L of BPA for 12 h and 24 h. The final DMSO concentration in all cell cultures was adjusted to 0.1% (v/v). Cell viability was determined using a CCK-8 commercial kit. Finally, the optical density was measured at 450 nm using a BioTek Epoch (BioTek Instruments, USA). The highest concentration of BPA that did not significantly affect cell viability was selected as the optimal concentration for subsequent experiments.

### 2.5 Cell apoptosis assay

TM3 cells were plated in 6-well plates at a density of 3×10^5^ cells/well and incubated for 24 h prior to treatment. Then, the cells were treated with 0, 20 and 40 μmol/L of BPA for 24 h, then gently digested with 0.5% trypsin (EDTA-free) and rinsed with 0.01 M phosphate buffer solution (PBS, 1×) twice. An Annexin V-FITC Apoptosis Detection Kit was used to characterize apoptosis. Cell pellets were re-suspended to a concentration of 5×10^5^ cells/mL at 500 μL binding buffer. One hundred microliters of the cell suspension was placed into a 5 mL flow-through tube, to which 5 µL of Annexin V/FITC was added and mixed well. The cells were then incubated in the dark at 25°C for 5 min, 5 μL propidium iodide and 400 µL PBS was added within 1 min before testing. Finally, the samples were assayed by flow cytometry (FACSCelesta, Becton Dickinson, USA). All data were analyzed using Flow.joX.10.0.7 software.

### 2.6 RNA extraction and RT-qPCR

TM3 cells were plated in 6-well plates at a density of 3×10^5^ cells/well and incubated for 24 h prior to treatment. The cells were divided into two groups and treated with 0, 20 μmol/L BPA for 24 h. Total RNA from TM3 cell samples and mouse testis tissues was extracted using TRIzol reagent. Total RNA was reverse transcribed into cDNA using the PrimeScript™RT Reagent Kit according to the manufacturer’s instructions, with gDNA Eraser, reverse transcription was performed using a 20 μL system set at 37°C for 15 min, 85°C for 5 s. The primer sets used for RT-qPCR is summarized in **Table 1**, which were constructed as span introns to avoid genomic DNA amplification. *Gapdh* was used as an internal reference, and mRNA expression was detected using the SYBR^®^ Green Realtime PCR Master Mix. The predegeneration was 95°C for 30 s with 1 cycle, and circular reaction was 95°C for 5 s, 55°C for 10 s, 72°C for 15 s for 40 cycles. Melting curve analysis was performed to determine the melting peaks to ensure that only a single product was amplified. All reactions were performed in triplicate. The 2^-△△Ct^ method was implemented for the quantitative analysis of the relative levels of mRNA expression, which were further normalized to the average level of the steadily expressed caretaker gene *Gapdh* (40).

**Table 1:** Primers sequences for the targeted genes in RT-qPCR.

Gene	Accession No.	Primer sequence (5'→3')	Product length(bp)
*Apoa1*	NM_009692.4	F: CAAAGACAGCGGCA GAGACT R: AGTTTTCCAGGAGATT CAGGTTCA	83
*Apoa*2	NM_013474.2	F: GAATCGCAGCACTGT TCCTA R: GTCTCTTAACCAAAGC TCCTTCC	107
*Apoc*3	NM_001289755.1	F: GAACAAGCCTCCAAG ACGGT R: GTTGGTTGGTCCTCA GGGTT	173
*Gapdh*	NM_001289726	F: GCCTCCTCCAATTCAACCCT R: CCCAATACGGCCAAATCCGT	145

### 2.7 Western blot

TM3 cells were plated in 6-well plates at a density of 3×10^5^ cells/well and incubated for 24 h prior to treatment. The cells were divided into two groups and treated with 0, 20 μmol/L BPA for 24 h. Cellular and testicular tissue proteins were extracted using lysates containing protease and phosphatase inhibitors, and the protein concentration in the extracts was measured using the Enhanced BCA Protein Assay Kit according to the manufacturer’s instruction. Dual Color SDS-PAGE Protein Sample Loading Buffer (6×) was added proportionally. The same amounts of protein were separated individually on a 10% SDS-PAGE gel and then transferred to PVDF membranes. QuickBlock™ Blocking Buffer was incubated at 25°C for 15 min, the membranes were incubated with primary antibodies at 4°C overnight and horseradish peroxidase-conjugated (1:5000) secondary antibodies at 25°C for 1 h sequentially. The two antibodies were diluted in the appropriate diluents as follows: APOA1 (1:1000) and ACTB (1:3000). The target bands were detected using BeyoECL Plus reagent. A Tanon 5200 luminescence imaging system (Tanon 5200, Yuanpinghao Biotechnology, China) and ImageJ 1.53a software (National Institute of Health, Bethesda, MD, USA) were used to visualize and digitize the immunoreactive bands, respectively (41). ACTB was used as an internal reference protein. Relative expression was derived from the grey value of the target gene and protein grey value/inner reference gene.

### 2.8 Immunofluorescence analysis

The testes were embedded in paraffin, and 6 μm thick paraffin sections were cut with a rotary microtome. Sections were dewaxed in xylene and rehydrated in a graded alcohol series. After blocking with 5% bovine serum albumin (BSA), the sections were incubated with anti-APOA1 (1:200) antibody and the corresponding secondary antibody (1:500). As a negative control, the anti-APOA1 antibody was replaced with PBS (1×). Microscopic examination was performed using a fluorescence microscope (Leica DM IL LED; Leica Camera, Germany). Three different fields of view were randomly selected for each group, and three images were quantitatively analyzed using ImageJ 1.53a software (41). For cellular immunofluorescence, TM3 cells were inoculated in 6-well plates with cell crawlers in advance, and 2×10^5^ cells were assessed per well and incubated for 24 h prior to treatment. The cells were divided into two groups and treated with 0, 20 μmol/L BPA for 24 h. The cells were rinsed three times with PBS (1×), fixed, stained, photographed, and analyzed as described above.

### 2.9 Oil red O staining and Filipin III staining

Frozen sections of 6 µm testis were fixed in 4% paraformaldehyde for 15 min and washed with distilled water thrice. The sections were stained with Oil Red solution for 10 min and washed with distilled water again. The sections were rinsed with 60% isopropanol for 2–30 s until the stroma was clear. It was then washed a further 2–5 times with water until there is no excess dye runoff. The sections were stained with hematoxylin for 5 min, washed with tap water until the nuclei turned blue, dried, and sealed with glycerol gelatin. The sections were visualized using an optical microscope (SOPTOP EX21, Ningbo Sunny Instruments, China). Three images were randomly selected from each group and quantitatively analyzed using ImageJ 1.53a software (41). For Oil Red O staining, TM3 cells were plated in 6-well plates at a density of 3×10^5^ cells per well and incubated for 24 h prior to treatment. The cells were divided into two groups and treated with 0, 20 μmol/L BPA for 24 h. Then, the culture medium was discarded and washed three times with PBS (1×), fixed, stained with oil red O, washed, re-stained with hematoxylin, washed, photographed, and analyzed as described above.

For Filipin III staining, the cells were treated in the same manner as in the Oil Red O experiment. Processed cell samples were fixed in 4% paraformaldehyde, washed with PBS (1×) and incubated with glycine (1.5 mg/mL PBS) at 25°C for 10 min to quench paraformaldehyde, and then rinsed again three times with PBS (1×). Afterwards, the cells were stained with 50 μg/mL Filipin III at room temperature for 30 min. The samples were rinsed three times with PBS (1×), and the fluorescence intensity of Filipin III was visualized using fluorescence microscopy. Filipin III had excitation maxima at 338 and 357 nm. In each experiment, three randomly selected fields per sample were visualized and captured. The resulting fluorescence was quantified using ImageJ 1.53a software and expressed as relative fluorescence intensity per cell (41).

### 2.10 Biochemical analysis

Testicular tissue samples were collected and homogenized using phosphate buffer (0.1 mol/L, pH 7.4), the samples were then pulverized and homogenized using a high throughput tissue grinder. The processed testes were cryo-centrifuged at 2500 rpm for 10 min at 4°C and the supernatant was retained for subsequent testing. The testis tissue supernatant was used to evaluate the levels of TC (detection limits: 0–19.39 mmol/L), HDL-C (detection limits: 0.09–2.50 mmol/L), and FC levels (detection limits: 0.055–4 mmol/L).

### 2.11 Testosterone measurement

TM3 cells were plated in 6-well plates at a density of 3×10^5^ cells/well and incubated for 24 h prior to treatment. The cells were divided into 4 groups, specifically the CON, BPA (20 μmol/L), 22-OH-Chol (30 μmol/L), and 22-OH-Chol (30 μmol/L) and BPA (20 μmol/L) co-treatment groups, with 3 replicates in each group, and were treated for 24 h. Mouse serum, TM3 cell culture supernatant, and testicular supernatant was measured using a highly sensitive ELISA detection kit according to the manufacturer’s instructions. The detection limit was less than 0.1 ng/mL, and the intra-assay coefficient of variation (CV%) was less than 10%. Absorbance was measured at 450 nm using a BioTek Epoch.

### 2.12 Statistical analysis

The data was shown with mean ± standard error mean (S.E.M) of at least three independent experiments and were performed in triplicate. For *in vivo* experiments, statistical analysis was conducted using data from 6 mice. GraphPad Prism (version 10.0) was used for graph visualization and data analysis. Differences between groups were analyzed using Student’s t-test or single-factor analysis of variance (one-way ANOVA). Differences with *P*-values of less than 0.05 were considered statistically significant.

## 3 Results

### 3.1 Effects of BPA exposure on testicular and serum testosterone levels

**Figure 1:**
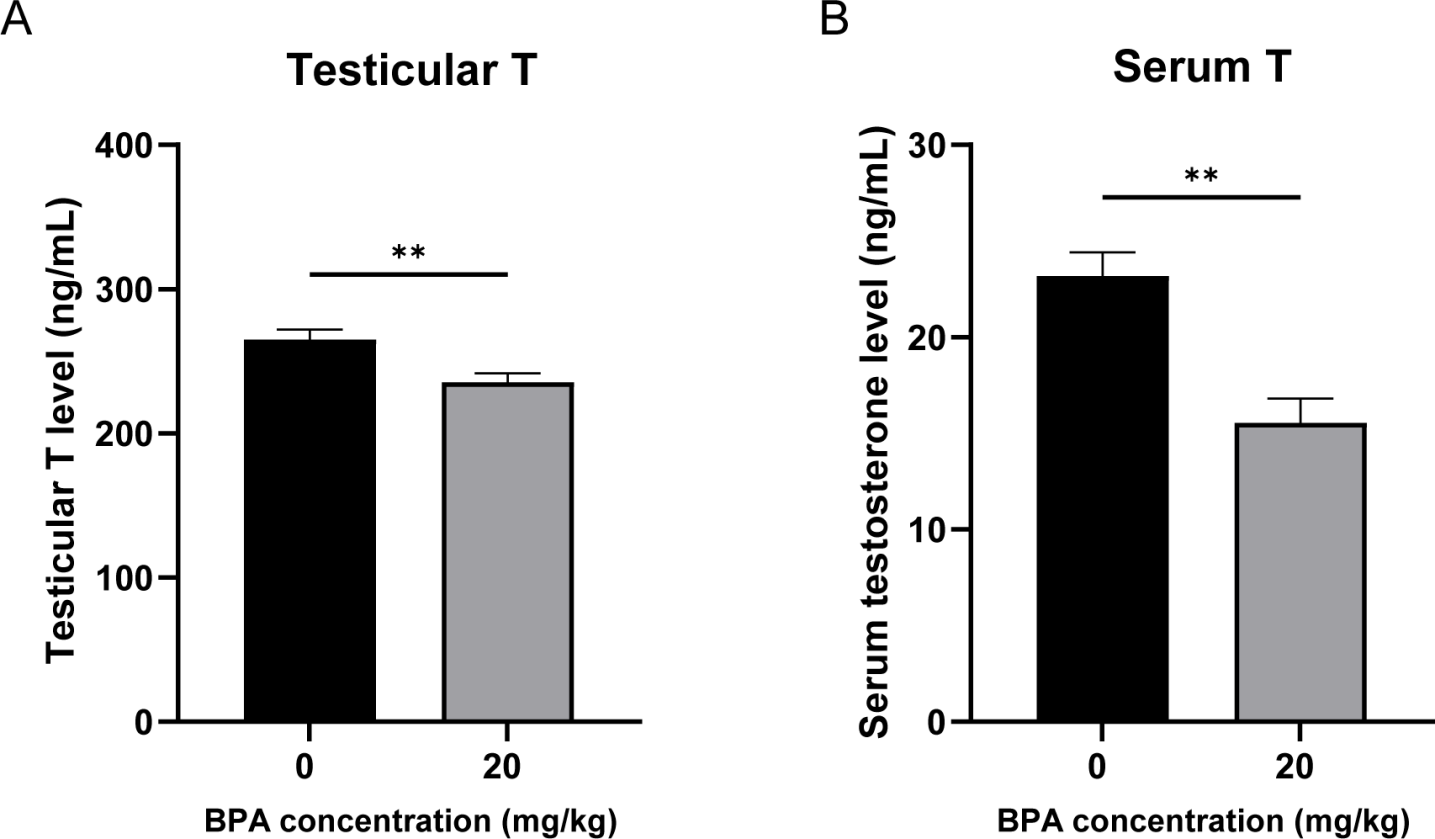
Effect of BPA exposure on testicular and serum testosterone levels. **(A)** Detection of mice **(A)** testicular and **(B)** serum testosterone levels after BPA treatment. All data were presented as means ± S.E.M (n=6). Differences were considered significant at *P* < 0.05. Asterisks indicate significant differences between CON and BPA treatment groups. **P < 0.01.

To investigate the effects of BPA exposure on serum and testicular testosterone synthesis in mice, testosterone levels were measured using ELISA. The results showed dramatic declines in serum and testicular testosterone levels in BPA-treated mice compared to those in the CON group (*P* < 0.01, **Figures 1A, B**). These results indicate that BPA exposure significantly inhibits testosterone synthesis and secretion in mice.

### 3.2 Effect of BPA exposure on lipid droplets, cholesterol, and HDL in mice testis

**Figure 2:**
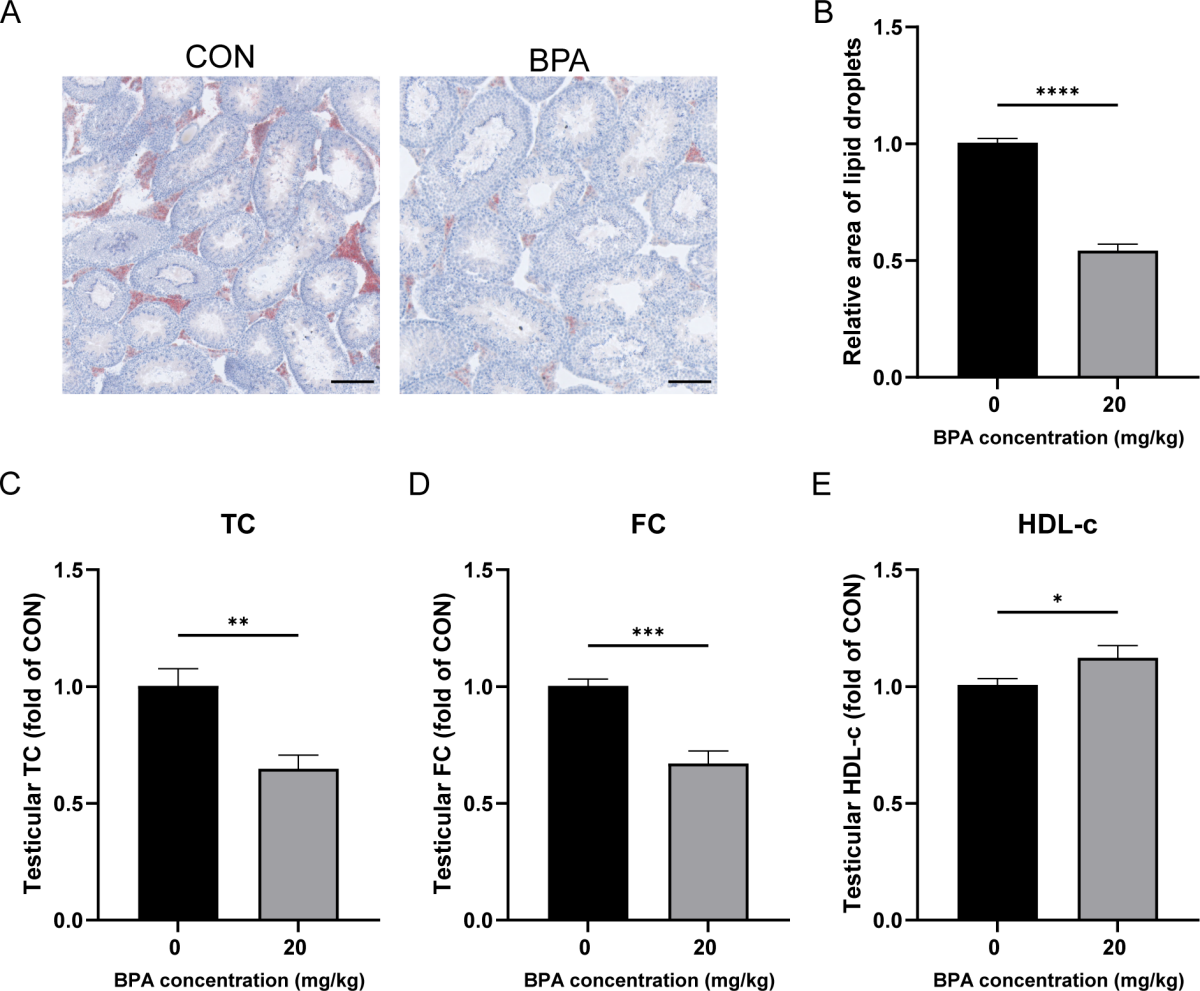
Effect of BPA exposure on lipid droplets, cholesterol, and HDL in mice testis. **(A)** Detection of relative accumulation of lipid droplets in testicular tissues treated with BPA, scale bar (100 μm). **(B)** Statistical analysis of area of lipid droplets results. Detection of levels of **(C)** TC, **(D)** FC, and **(E)** HDL-c in testis after BPA treatment. All data were presented as means ± S.E.M (n=6). Differences were considered significant at *P* < 0.05. Asterisks indicate significant differences between CON and BPA treatment groups. *P < 0.05, **P < 0.01, ***P < 0.001, ****P < 0.0001.

Lipid droplets are the primary storage sites for neutral lipids in cells. Because the accumulation of lipid droplets in testicular tissue is correlated with cholesterol levels, Oil Red O staining was used to study the accumulation of lipid droplets in mouse testicular tissue. Oil Red O staining of testicular tissue revealed that lipid droplet accumulation was significantly reduced in the BPA-treated group compared to that in the CON group (*P* < 0.0001, **Figures 2A, B**). Compared with the CON group, testicular TC and FC levels were significantly decreased following BPA exposure (*P* < 0.01 and *P* < 0.001, respectively, **Figures 2C, D**). Testicular HDL-C levels significantly increased following BPA exposure (*P* < 0.05, **Figure 2E**). These results suggest that the exposure to BPA may enhance RCT, reduce cholesterol levels and lipid droplet accumulation, and ultimately inhibit testosterone synthesis and secretion in mouse testes.

### 3.3 Effects of BPA exposure on the expression of genes associated with RCT in mice testis

**Figure 3:**
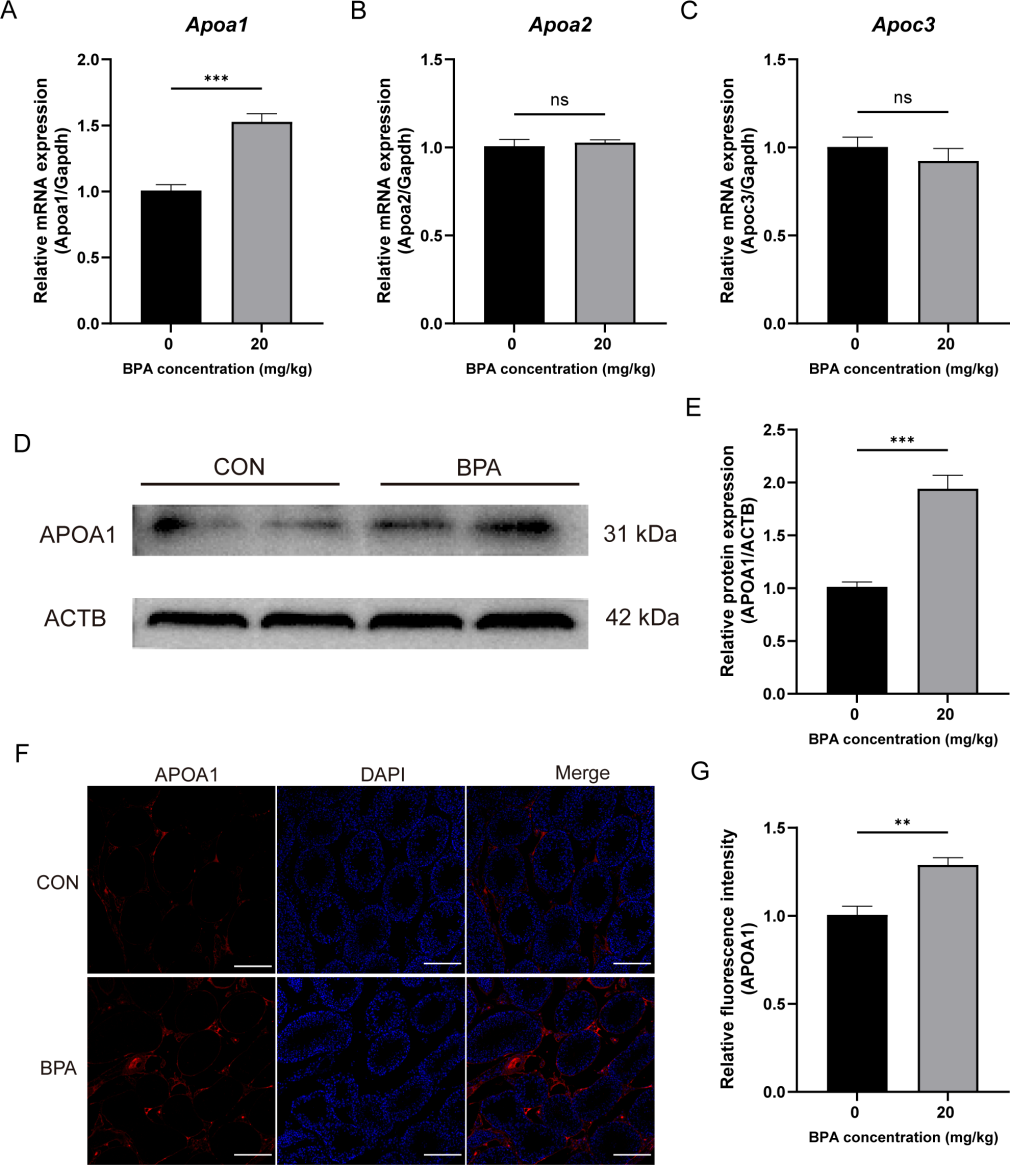
Effect of BPA exposure on the expression of genes associated with RCT in the mice testis. **(A–C)** Detection of relative expression levels of genes to *Apoa1*, *Apoa2*, and *Apoc3* in testicular tissues relative to the levels of the housekeeping gene *Gapdh*. **(D)** Detection of relative protein expression of APOA1 in testicular tissues. **(E)** Ratio of APOA1 to ACTB was determined from the densities of the immunoreactive bands, and the results are shown as a bar graph. **(F)** Detection of relative protein expression of APOA1 in testicular tissues, scale bar (200 μm). **(G)** Statistical analysis of fluorescent quantitative results. All data were presented as means ± S.E.M (n=6). Differences were considered significant at *P* < 0.05. Asterisks indicate significant differences between CON and BPA treatment groups. ***P < 0.001, **P < 0.01, ns *P* > 0.05.

To investigate the relationship between the inhibition of testosterone synthesis and RCT in mice following BPA exposure, the mRNA expression of *Apoa1*, *Apoa2*, and *Apoc3* in the testicles was detected using RT-qPCR. Following BPA exposure, *Apoa1* was significantly increased (*P* < 0.001, **Figure 3A**), whereas there was no significant difference in *Apoa2* and *Apoc3* compared with that of the CON group (**Figures 3B, C**). Western blotting revealed that APOA1 protein levels were significantly upregulated (*P* < 0.001, **Figures 3D, E**). The IF results showed that the fluorescence intensity of APOA1 in the BPA-treated group was significantly higher than that in the CON group (*P* < 0.001, **Figures 3F, G**). These results suggest that exposure to BPA enhances the RCT process in testicular tissues by up-regulating *Apoa1*, which in turn inhibits testosterone synthesis and secretion.

### 3.4 Effect of BPA exposure on TM3 cell viability

**Figure 4:**
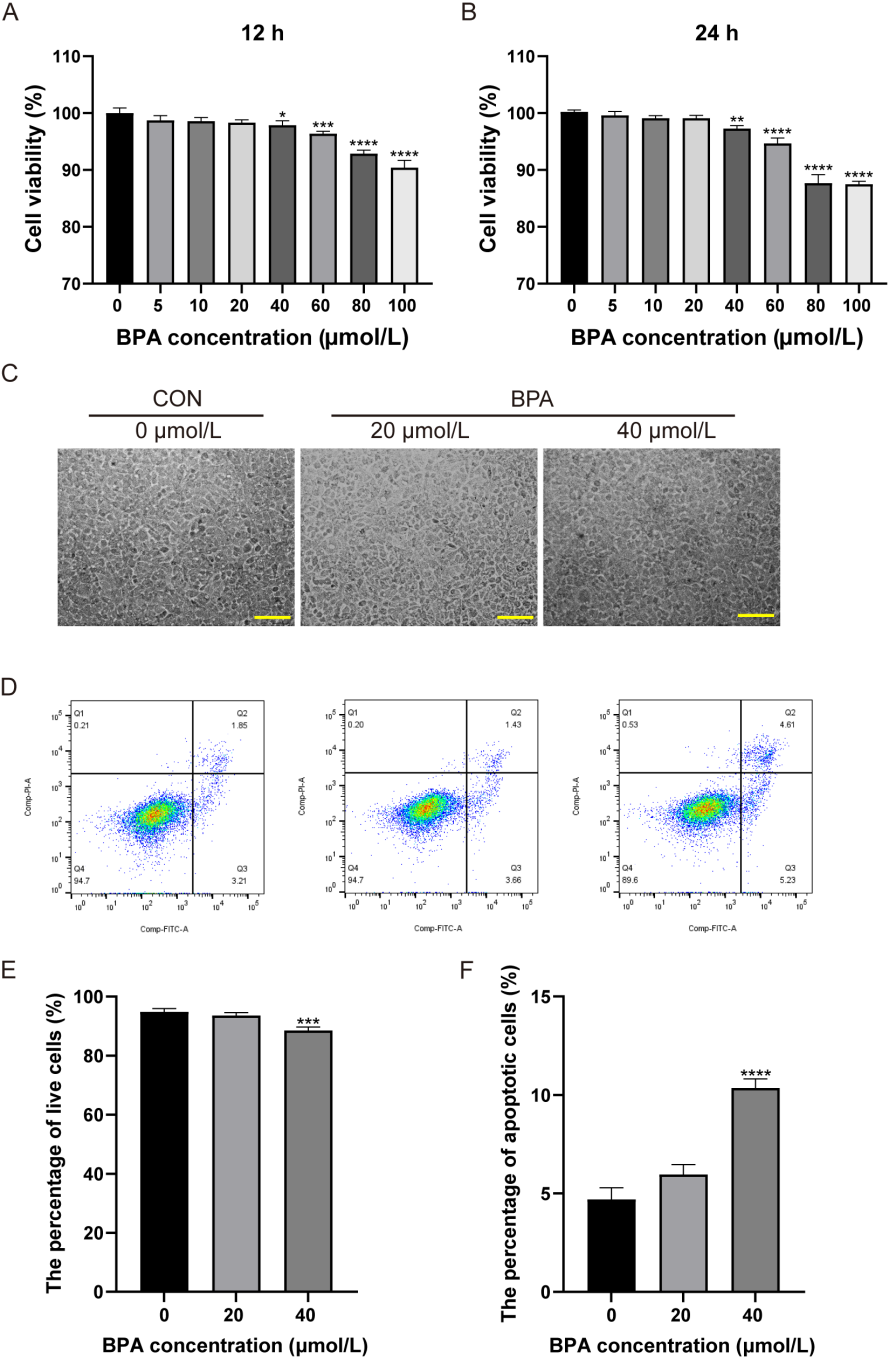
Determination of the optimal BPA treatment concentration in TM3 cells. Effects of different concentrations of BPA (0, 5, 10, 20, 40, 60, 80, or 100 μmol/L) on TM3 cell viability after **(A)** 12 h and **(B)** 24 h treatment. **(C)** Effects of 0, 20, and 40 μmol/L BPA on the growth status of TM3 cells after 24 h, scale bar (200 μm). **(D)** Detection of apoptosis in TM3 cells after 0, 20, and 40 μmol/L BPA treatment. Q1: necrotic cells and cell fragments, Q2: late apoptotic, Q3: normal cells, Q4: early apoptotic cells. **(E)** Percentage of viable cells in the 0, 20, and 40 μmol/L BPA groups. **(F)** Percentage of apoptotic cells in 0, 20, and 40 μmol/L BPA groups. All data were presented as means ± S.E.M of three independent experiments. Differences were considered significant at *P* < 0.05. Asterisks indicate significant differences between 0 μmol/L (CON) and BPA treatment groups at different concentrations. *P < 0.05, **P < 0.01, ***P < 0.001, ****P < 0.0001.

The CCK-8 assay was performed using different BPA concentrations for 12 and 24 h to verify the toxicity of BPA in TM3 cells. The data indicated that 40 μmol/L BPA significantly reduced the cell viability at 12 or 24 h compared to the group that received 0 μmol/L (*P* < 0.05 and *P* < 0.01, respectively; **Figures 4A, B**), cell viability was reduced by 2.11% after 12 h of BPA treatment and by 2.7% after 24 h of exposure. Hence, BPA exhibited a concentration-dependent inhibitory effect on the cell viability of TM3 cells at 12 and 24 h (**Figures 4A, B**). In addition, the status of TM3 cells cultured in 0, 20, and 40 μmol/L BPA for 24 h was observed. The cells in the 0 and 20 μmol/L BPA groups grew in a shuttle or polygonal shape with uniform distribution and good adherence to the wall, the 40 μmol/L BPA group showed no significant difference in cell density compared to the other two groups, although a few cells exhibited morphological changes (**Figure 4C**).

### 3.5 Effect of BPA exposure on apoptosis levels in TM3 cells

In order to further screen the optimal concentration of BPA, this study examined the apoptosis of cells treated with 0, 20, and 40 μmol/L BPA for 24 h. The apoptosis rate was not significantly affected in the 20 μmol/L BPA group compared to that of the CON group, and apoptosis was significantly increased (5.3%) in the 40 μmol/L BPA group compared to that of the CON group, and the type of apoptosis observed was primarily late apoptosis (*P* < 0.0001, **Figures 4D, F**); the percentage of viable cells in the 40 μmol/L BPA group was significantly lower than that in the CON group (*P* < 0.001, **Figures 4D, E**). The above results indicated that 20 μmol/L BPA treatment for 24 h had no significant effect on the cell viability of TM3 cells and was selected for subsequent experiments.

### 3.6 Effects of BPA exposure on testosterone synthesis in TM3 cells

**Figure 5:**
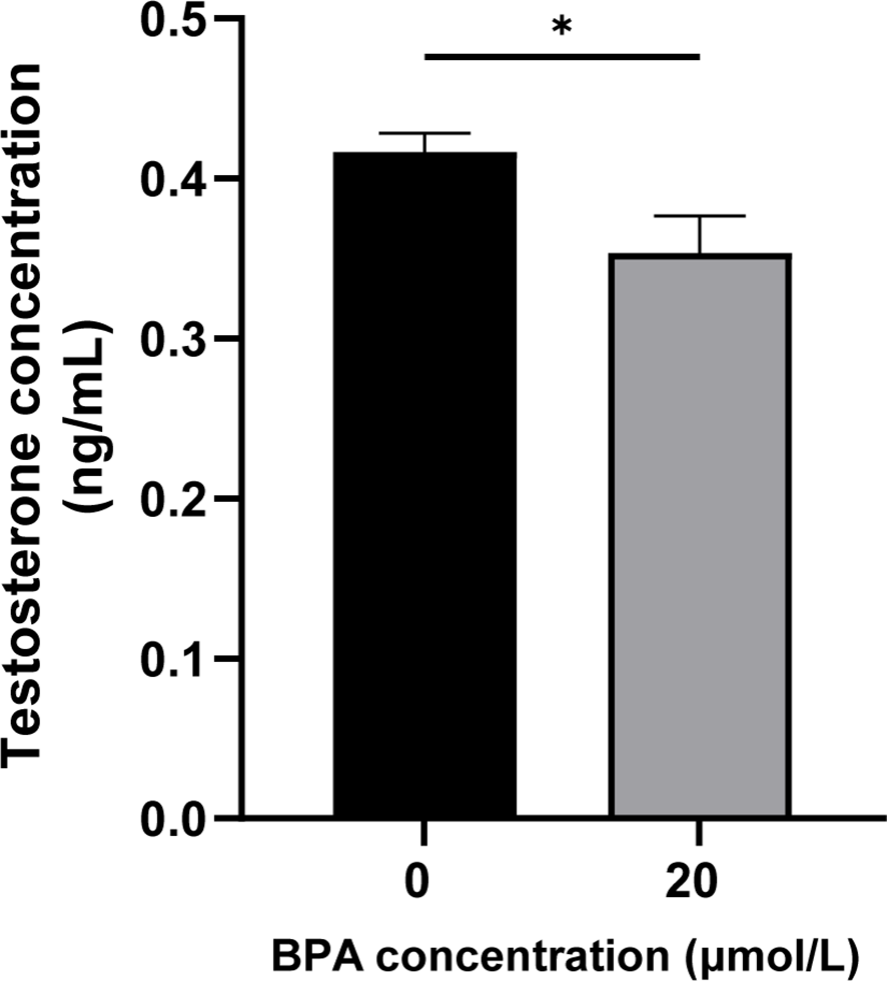
Effect of BPA exposure on testosterone synthesis in TM3 cells. All data were presented as means ± S.E.M of three independent experiments. Differences were considered significant at *P* < 0.05. Asterisks indicate significant differences between CON and BPA treatment groups. *P < 0.05.

To explore the effect of 20 μmol/L BPA on testosterone synthesis in TM3 cells, the testosterone secretion level was examined in the cellular supernatant. The results showed that testosterone levels in the cellular supernatant were significantly lower in the BPA-treated group than in the CON group (*P* < 0.05; **Figure 5**). The results suggest 20 μmol/L BPA treatment significantly inhibited testosterone secretion level in TM3 cells.

### 3.7 Effects of BPA exposure on the expression of genes involved with RCT in TM3 cells

**Figure 6:**
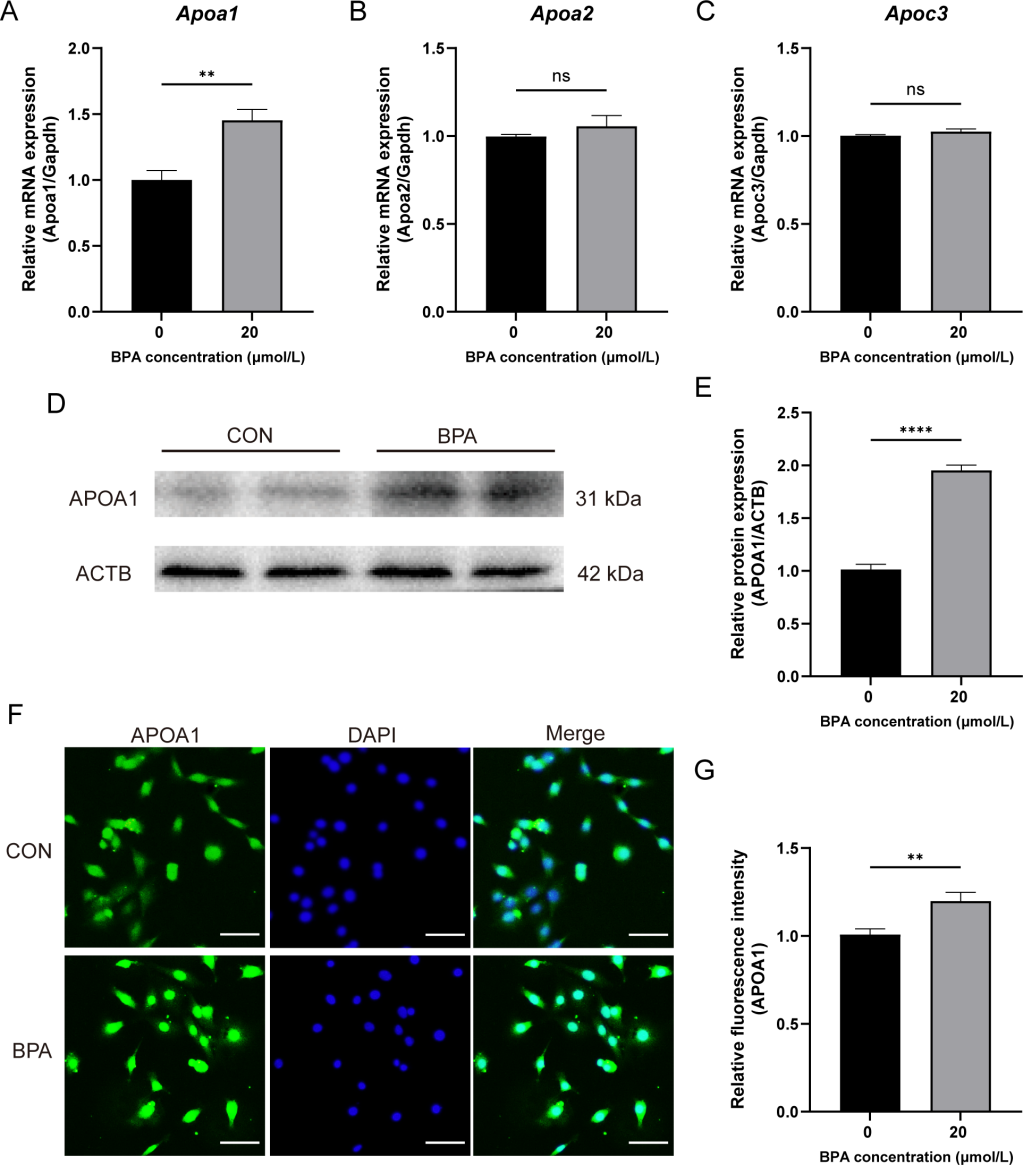
Effect of BPA exposure on the expression of genes involved with RCT in TM3 cells. **(A–C)** Detection of relative expression levels of genes to *Apoa1*, *Apoa2*, and *Apoc3* in the TM3 cells relative to the levels of the housekeeping gene *Gapdh*. **(D)** Detection of relative protein expression of APOA1 in TM3 cells. **(E)** Ratio of APOA1 to ACTB was determined from the densities of the immunoreactive bands, and the results are shown as a bar graph. **(F)** Detection of relative protein expression of APOA1 in TM3 cells, scale bar (50 μm). **(G)** Statistical analysis of fluorescent quantitative results. All data were presented as means ± S.E.M of three independent experiments. Differences were considered significant at *P* < 0.05. Asterisks indicate significant differences between CON and BPA treatment groups. **P < 0.01, ****P < 0.0001, ns *P* > 0.05.

To investigate the relationship between the inhibition of testosterone synthesis and RCT after BPA exposure, this study detected changes in the mRNA expression of *Apoa1*, *Apoa2*, and *Apoc3* in TM3 cells exposed to BPA for 24 h, using RT-qPCR. The mRNA expression level of *Apoa1* gene was significantly increased in the BPA-treated group compared to that of the CON group (*P* < 0.01, **Figure 6A**), whereas there was no significant difference in the mRNA expression of *Apoa2* and *Apoc3* genes compared to that of the CON group (**Figures 6B, C**). Western blotting results showed that the APOA1 protein level was significantly increased after BPA exposure (*P* < 0.01, **Figures 6D, E**), and the IF results showed that the intracellular APOA1 fluorescence intensity was significantly stronger after 24 h of BPA exposure than in the CON group (*P* < 0.01, **Figures 6F, G**). Taken together, these results suggested that upregulated *Apoa1* gene expression level may be associated with decreased testosterone synthesis and secretion in 20 μmol/L BPA treatment TM3 cells.

### 3.8 Effects of exposure to BPA on cholesterol and lipid droplets in TM3 cells

**Figure 7:**
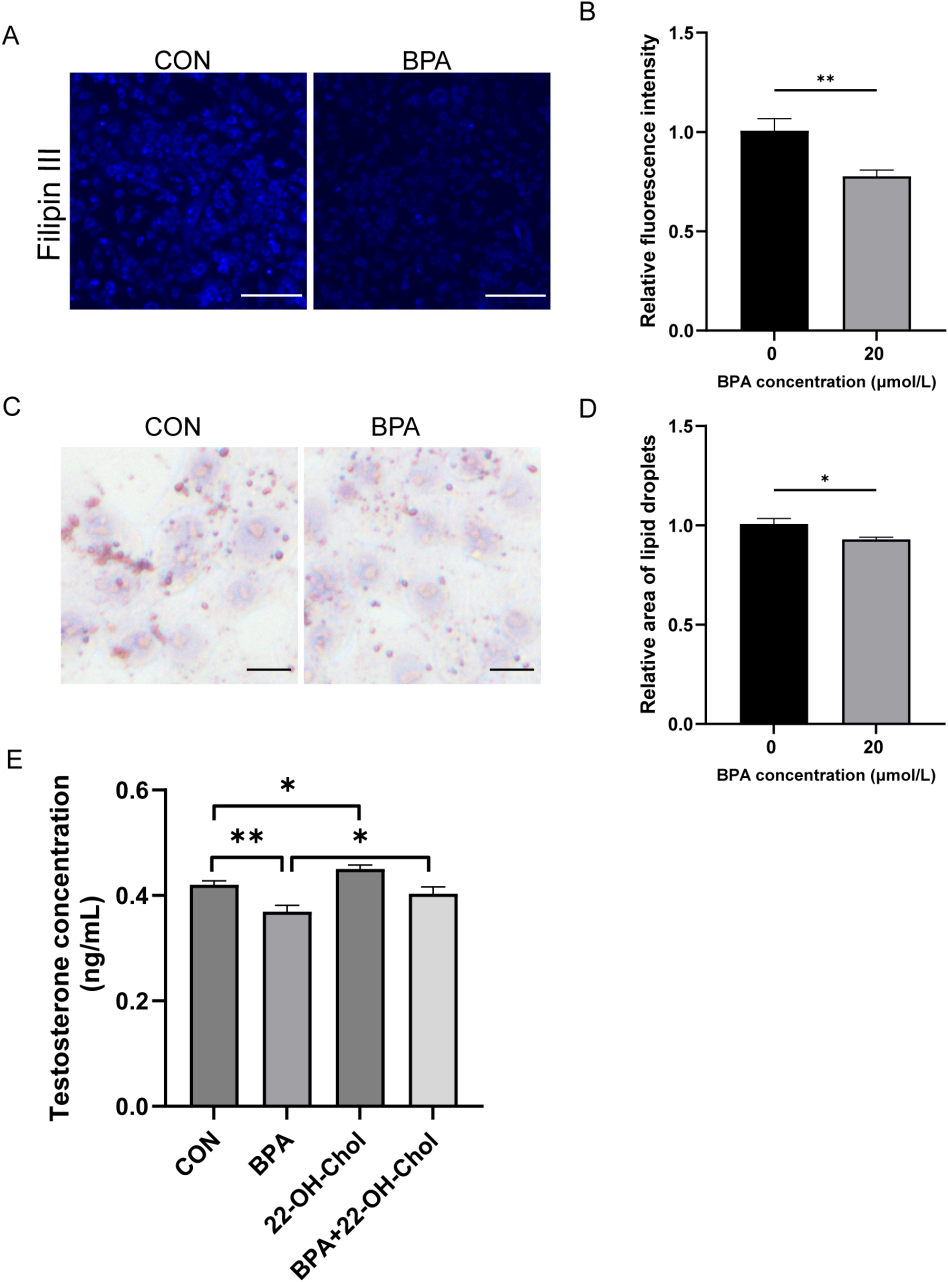
Effect of BPA exposure on cholesterol and lipid droplets in TM3 cells. **(A)** Detection of relative content of free cholesterol in TM3 cells treated with BPA for 24 h, scale bar (50 μm). **(B)** Statistical analysis of fluorescent intensity results. **(C)** Detection of relative content of lipid drops in TM3 cells treated with BPA for 24 h, scale bar (50 μm). **(D)** Statistical analysis of area of lipid droplets results. **(E)** Detection of testosterone levels in the culture media of TM3 cells treated with 22-OH-Chol and BPA for 24 h. All data were presented as means ± S.E.M of three independent experiments. Differences were considered significant at *P* < 0.05. Asterisks indicate significant differences between different groups. *P < 0.05, **P < 0.01.

This study investigated whether BPA inhibits testosterone synthesis and secretion by affecting cholesterol levels. After the TM3 cells were exposed to BPA for 24 h, cholesterol levels were detected by Filipin staining. Compared to that of the CON group, the level of FC was significantly reduced (*P* < 0.01, **Figures 7A, B**). Since the accumulation of lipid droplets in the cells correlated with a reduction in the available cholesterol pool, the accumulation of lipid droplets in TM3 cells after BPA treatment was further investigated. Oil Red O staining revealed that the number of lipid droplets was significantly lower than that in the CON group (*P* < 0.05, **Figures 7C, D**). To further validate whether changes in FC content mediates the effect of BPA on synthesis of testosterone in TM3 cells, the cells were treated with 22-OH-Chol (30 μmol/L) in the presence or absence of BPA for 24 h. The exogenous addition of 22-OH-Chol promoted testosterone synthesis (*P* < 0.05) and alleviated the inhibitory effect of BPA (*P* < 0.05, **Figure 7E**). These results suggest that BPA inhibits testosterone synthesis by reducing FC levels in TM3 cells.

## 4 Discussion

Numerous studies have focused on the hypothalamic-pituitary-gonadal axis following BPA exposure, whereas few studies have revealed the inhibition of testosterone synthesis and secretion from the perspective of lipid metabolism. Cholesterol is a raw material for testosterone synthesis, and the RCT process is directly related to the amount of cholesterol in the testes. This study aimed to investigate whether a reduction in testosterone synthesis is associated with changes in cholesterol following BPA exposure *in vivo* and *in vitro*. The results showed that TM3 cell survival progressively decreased with increasing BPA doses, indicating dose-dependent damage to cells by BPA. BPA exposure significantly increased mRNA expression and protein levels of *Apoa1*, and significantly reduced cholesterol levels and lipid droplet accumulation. Exogenous supplemented with 22-OH-Chol promoted testosterone synthesis and alleviated the inhibitory effects of BPA on testosterone synthesis in TM3 cells. These results demonstrated that BPA attenuates testosterone production, at least in part, by enhancing APOA1 expression, which enhances the RCT process and ultimately leads to a decrease in cholesterol.

The reproductive system is the most sensitive to environmental risk factors. BPA has typical environmental estrogenic effects that damage endogenous hormone synthesis through its receptors and cause abnormalities in the reproductive endocrine system of animals (39, 42). The results of this study indicate that BPA significantly reduces testosterone synthesis and secretion in TM3 cells. Similarly, Goncalves et al. (43) determined that BPA decreased testosterone production in a dose-dependent manner through the impairment of steroidogenesis in TM3 cells (43). Several studies have reported that BPA impairs testosterone synthesis in testicular Leydig R2C and MA-10 cells (44, 45). Treatment with BPA also decreases testosterone synthesis by 25% in adult rat Leydig cells (46). Numerous studies have shown that testosterone production in rodents is significantly attenuated by BPA treatment (43, 46, 47). In agreement with the above reports, the current data revealed that BPA exposure severely decreased testosterone production in TM3 cells, mouse serum, and testis.

Previous studies have shown that BPA induces the expression of steroidogenic genes by activating c-Jun phosphorylation, leading to a significant decrease in the testosterone/estrogen ratio in BPA-treated male SD rats (44, 48). Hinfray et al. (49) demonstrated that BPA may affect sex hormone synthesis secretion by regulating the expression of steroidogenesis-related genes (49). In addition, BPA affects hormone levels through various mechanisms, including the hypothalamic-pituitary-gonadal axis in mice (50). However, most previous studies have focused on testosterone synthesis-related genes and neurohumoral regulation, and few studies have reported the direct effect of BPA on cholesterol levels in Leydig cells and their effect on testosterone synthesis and secretion. The results of this study showed that the level of FC in TM3 cells and mouse testes significantly decreased after BPA exposure. 22-OH-Chol, an intermediate in the synthesis of pregnenolone by a cholesterol side-chain cleavage enzyme, can be added exogenously to isolated Leydig cells for testosterone synthesis (51). It has been shown that melatonin could inhibit the synthesis of testosterone in Leydig cells of roosters by reducing free cholesterol content, and the exogenous addition of 22-OH-Chol could promote synthesis of testosterone and alleviated the inhibitory effect of melatonin on synthesis of testosterone in Leydig cells (52). This is consistent with our findings, as the testosterone levels in the 22-OH-Chol and BPA co-treatment group were not significantly different from those in the CON group and were significantly increased compared to that of the BPA alone group. Cholesterol accumulates in the cytoplasm in its free form or is stored in lipid droplets in its ester form (53). Combined with the Oil Red O results, the disappearing cholesterol was not stored in the lipid droplets by conversion to CEs, thereby increasing lipid droplet accumulation. In contrast, the lipid droplet levels were significantly reduced in the BPA group. Cholesterol homeostasis is maintained by supply and consumption through the lipoprotein transport pathways and *de novo* synthesis (54). This suggests that BPA may lead to reduced testosterone synthesis and secretion by disrupting cholesterol homeostasis.

APOA1 is a plasma apolipoprotein comprising the major protein component of HDL, which is involved in RCT from the periphery to the liver. The RCT is considered the only pathway that reduces cholesterol load in the peripheral compartment by increasing its flux towards the liver, where it can be catabolized (35). It has been shown that the impaired expression of *Apoa1* inhibits cholesterol efflux and HDL production, causing disturbances in cholesterol metabolism in the body (55). Increased plasma APOA1 levels promote RCT and lower plasma cholesterol levels. The results of the present study indicated that BPA exposure results in a significant increase in the mRNA expression and protein levels of the *Apoa1* gene, along with a significant increase in HDL-C levels and a significant decrease in FC and TC levels in the testis. This suggests that BPA enhances the RCT process by upregulating *Apoa1* expression, leading to a decrease in intracellular FC content, which is similar to the findings of Zhang et al. (19). This finding has been confirmed in other studies; for example, cholesterol levels in the testes of male Kunming rats were significantly suppressed after being fed 25 mg/kg BPA by gavage daily for four consecutive weeks (56). Some studies have shown that middle-aged male mice exhibit obesity, glucose intolerance, dyslipidemia, and hepatic accumulation of triglycerides and cholesterol after BPA exposure. Liver cells from BPA-exposed mice showed significantly increased expression of genes related to lipid synthesis, causing hepatic lipid accumulation (57). Therefore, the present study suggests that BPA reduces testicular FC and TC levels by increasing RCT, thereby decreasing testosterone levels.

HDLs are the major vehicles of RCT (58, 59). Elevated serum APOA1 levels promote RCT and lower blood cholesterol levels; conversely, APOA1 deficiency has the opposite effects (60). Using *Apoe*-deficient (*Apoe* ^-/-^) and double-deficient (*Apoe* ^-/-^ and *Apoa1* ^-/-^) mouse models, it was demonstrated that only *Apoa1* efficiently reduced cholesterol accumulation in lesions *in vivo* (61). From the present study, BPA exposure significantly upregulated *Apoa1* mRNA and protein levels, whereas there was no significant effect on *Apoa2* and *Apoc3* via *in vivo* and *in vitro* experiments. Similarly, Zabalawi et al. (62) demonstrated a significantly higher aortic esterified cholesterol and a major accumulation of skin cholesterol after consuming a high-fat diet in double-deficient (*LDLr* ^-/-^ and *Apoa1* ^-/-^) mice than in *LDLr* ^-/-^ mice. Although comparable amounts of other apolipoproteins, including Apolipoprotein A4 (APOA4), APOA2, and C apolipoproteins, were detected in both groups of mice (62, 63), these proteins do not replace the role of APOA1 in RCT to promote the efficient transfer of excess cholesterol from peripheral cells and do not facilitate efficient cholesterol efflux from lesions. This indicates that APOA1 is much more important in the RCT process than other proteins.

## 5 Conclusion

In summary, this study confirmed that BPA exposure enhances APOA1-mediated RCT, which leads to a decrease in cholesterol, the raw material for testosterone synthesis in mouse testes, and ultimately leads to a decrease in testosterone synthesis. This study provides new insights into the mechanism through which BPA inhibits testosterone by disrupting cholesterol transport.

## Data Availability

The raw data supporting the conclusions of this article will be made available by the authors, without undue reservation.
